# Whipworm infection remodels the gut microbiome ecosystem and compromises intestinal homeostasis in elderly patients revealed by multi-omics analyses

**DOI:** 10.3389/fcimb.2025.1663666

**Published:** 2025-08-29

**Authors:** Benguang Zhang, Zhaoan Sheng, Cancan Bu, Longjiang Wang, Wenxiang Lv, Yongbin Wang, Yan Xu, Ge Yan, Maoqing Gong, Lijuan Liu, Wei Hu

**Affiliations:** ^1^ State Key Laboratory of Genetic Engineering, Ministry of Education Key Laboratory for Biodiversity Science and Ecological Engineering, Ministry of Education Key Laboratory of Contemporary Anthropology, School of Life Science, Fudan University, Shanghai, China; ^2^ Shandong Institute of Parasitic Diseases, Shandong First Medical University and Shandong Academy of Medical Sciences, Jining, Shandong, China; ^3^ School of Public Health, Shandong First Medical University and Shandong Academy of Medical Sciences, Jining, Shandong, China; ^4^ Department of Pathogenic Biology, Jining Medical University, Jining, Shandong, China; ^5^ State Key Laboratory of Reproductive Regulation and Breeding of Grassland Livestock, School of Life Sciences, Inner Mongolia University, Hohhot, China; ^6^ National Institute of Parasitic Diseases, Chinese Center for Disease Control and Prevention (Chinese Center for Tropical Diseases Research), Key Laboratory of National Health Commission on Parasite and Vector Biology, World Health Organization (WHO) Collaborating Centre for Tropical Diseases, National Center for International Research on Tropical Diseases, Ministry of Science and Technology, Shanghai, China

**Keywords:** whipworm, gut microbiota, metabolomics, multi-omics, metagenomic

## Abstract

**Introduction:**

Whipworm (*Trichuris trichiura*) coexists with symbiotic microbiota in the gastrointestinal ecosystem. There is a paucity of data on the association between whipworm infection and the gut microbiota composition in elderly individuals. This study was designed to investigate changes in gut microbiota and function and its metabolite profile in patients with whipworm infection.

**Methods:**

We used 16S rRNA gene sequencing to identify microbial signatures associated with whipworm infection. Subsequently, shotgun metagenomic sequencing revealed functional changes that highlighted disruptions in microbial gene expression and metabolic pathways influencing host health. Ultraperformance liquid chromatography-mass spectrometry metabolomics was used to characterize whipworm infectioninduced metabolic perturbations and elucidate metabolite dynamics linked to microbial activity. Collectively, this multi-omics approach deciphered structural, functional, and metabolic remodeling of the gut ecosystem that distinguished whipworm-infected patients from healthy controls.

**Results:**

Analyses of the gut microbiome in patients with whipworm infection revealed significantly increased observed species richness and ACE indices, along with an enrichment of *Prevotella 9*-driven enterotypes. Additionally, metagenomic and metabolomic analyses indicated enrichment in metabolic pathways related to amino acid, energy and carbohydrate metabolism. Metabolic network analysis further suggested that the upregulated *Prevotella copri* and *Siphoviridae* sp. were positively correlated with elevated levels of myristic acid and DL-dipalmitoylphosphatidylcholine.

**Conclusion:**

These findings suggest that whipworm infection significantly remodels the gut microbiome ecosystem and compromises intestinal homeostasis.

## Introduction

Whipworm (*Trichuris trichiura*), a soil-transmitted helminth, is a persistent yet neglected tropical disease of global health concern. Approximately 464 million individuals worldwide are thought to be infected, particularly children, with endemic transmission concentrated in regions characterized by inadequate sanitation infrastructures and socioeconomic underdevelopment (Pullan et al., 2014). Whipworm infections can lead to a variety of clinical symptoms, including *Trichuris* dysentery syndrome, anemia, stunted growth, malnutrition, diarrhea, and cognitive impairments (Stephenson et al., 2000). These conditions present considerable public health challenges in areas affected by whipworm.

The majority of gut microbiota are beneficial and crucial for host physiology by contributing to digestion, immune system development, behavioral modulation, and by influencing metabolic and cardiovascular health (Lin et al., 2025). Both helminth and the gut microbiota compete for similar ecological niches within the host, which may lead to shifts in the microbial ecosystem. Emerging evidence suggests that many helminth infections, including whipworm, significantly alter the composition and diversity of the gut microbiota (Gordon et al., 2020; Lee et al., 2014; Rosa et al., 2018). Moreover, the presence of helminth, such as whipworm, can trigger the host’s immune response, further disrupting the balance between the microbiota and the host (Schytz et al., 2024). These disruptions may compromise intestinal barrier integrity, dysregulate immune function, and alter metabolic pathways. Whipworm infection specifically enriches microbial taxa that either facilitate parasite persistence or exacerbate pathogenesis. The effects of helminth colonization on the human gut microbiota has yielded conflicting reports on microbial diversity and taxon-specific alterations. While some studies have suggested that helminth infection significantly changes the microbial composition of the gut microbiota, other studies have reported these microbial changes as minimal, likely due to confounding factors such as geographical heterogeneity in host populations, parasite species-specific effects (*Trichuris*, hookworm, *Ascaris*, *Strongyloides*, *Schistosoma*) (Cooper et al., 2013) and methodological variations in sequencing platforms and bioinformatic pipelines. Most current studies use 16S rRNA sequencing, highlighting the need for large-scale metagenomic studies that integrate infection status and geographically matched controls to clarify these complex interactions.

Based on our preliminary research, a notably high prevalence of whipworm infections among individuals aged over 60 was found in Shandong Province (Xu et al., 2023). This trend significantly differed from the typical epidemiology seen in developing countries, where children usually show the highest rates of trichuriasis. Given this contrast, our study focused on understanding how *Trichuris trichiura* infection altered the gut microbiome in elderly populations to provide insights into age-specific effects of the infection. We combined 16S ribosomal RNA sequencing, metagenomic profiling, and ultraperformance liquid chromatography-mass spectrometry (UHPLC-MS) to comprehensively characterize both the phylogenetic structure of the gut microbiota and the associated bioactive metabolomic profiles in individuals with varying degrees of infection. The 16S sequencing allowed us to profile microbial taxonomy and identify infection-associated signatures. Shotgun metagenomics was used to elucidate potential functional and pathway changes influencing host health. Metabolomics was used to characterize infection-induced metabolic perturbations linked to microbial activity. By integrating these methods, our study aimed to provide a comprehensive understanding of the manner by which whipworm infection influences gut microbiota composition, function, and metabolic activity, particularly in the elderly. Ultimately, we sought to identify key microbial and metabolic biomarkers to serve as diagnostic tools or therapeutic targets to help improve health outcomes in elderly individuals affected by whipworm infection.

## Methods

### Sample collection

Fecal samples were collected from participants from 2020 to 2023 in Lanshan District, Rizhao City, Shandong Province, China. For each participant, key demographic and health-related information was recorded, including age, gender, height, weight, dietary habits, and medication history. The participants were also confirmed to be undergoing deworming for the first time, and had not received any medication prior to the administration of anthelmintics. Fecal samples were collected with the participant’s consent before the anthelmintics were administered.

### Sample processing

All participants with gastrointestinal disorders, autoimmune diseases, cardiovascular diseases, malignancies, or those who had taken antibiotics, anti-parasitic medications or glucocorticoids within the last 3 months were excluded. Fecal samples were collected from each participant and examined for *T. trichiura* eggs using a standardized modified Kato-Katz thick smear technique (Ting-Jun et al., 2018) with two smears per stool sample. *T. trichiura* infection was confirmed by two parasitology specialists who ensured that only *T. trichiura* eggs and no other parasites were present in one or more smears. The infection intensity was categorized as light, moderate, or heavy based on the World Health Organization (WHO) guidelines (Committee, 2002). The basic information and examination results of all participants were recorded in the China Information Management System of Parasitic Diseases Prevention and Control. Each fecal sample was divided into three parts after collection, transported to the laboratory on dry ice and finally stored at -80°C.

### DNA extraction, library preparation, 16S rRNA gene sequencing, and analysis

Genomic DNA was extracted from stool samples using a TIANamp stool DNA kit (TIANGEN, China) in accordance with the manufacturer’s protocol, which incorporated mechanical lysis with bead agitation and chemical lysis with specialized buffers to ensure optimal DNA yields. The V3–V4 hypervariable regions of the bacterial 16S rRNA gene were PCR-amplified using primers 341F (5′-CCTAYGGGRBGCASCAG-3′) and 806R (5′-GGACTACNNGGGTATCTAAT-3′). Sequencing libraries were constructed using the Rapid Plus DNA Lib prep kit for Illumina (ABclonal Biotechnology, China), incorporating unique barcodes for multiplexing and following the manufacturer’s protocol. Sequencing was conducted on an Illumina NovaSeq 6000 (Illumina, USA) system using standard Illumina procedures. Initial quality control and adapter trimming were performed using Trimmomatic (v0.33) to remove low-quality bases and adapter sequences. Primer sequences were identified and excised using cutadapt (v1.9.1), yielding high-quality clean reads. The clean reads were then processed for denoising with the DADA2 algorithm implemented in QIIME *2* (v2020.6), which produced error profiles, merged paired-end reads, and filtered chimeric sequences. This produced non-chimeric amplicon sequence variants (ASVs), which constituted the final feature table. The ASV table was then used for the following downstream analyses: microbial feature characterization through taxonomic assignment of ASVs; assessment of alpha and beta diversity; identification of differentially abundant taxa between groups; inference of microbial co-occurrence patterns using correlation network analysis; and functional profiling via metagenomic predictions of the 16S data. Detailed methodologies and specific statistical parameters for each analysis are described in the corresponding results sections.

### Metagenome assembly and functional annotations

Metagenomic sequencing was performed on an Illumina NovaSeq 6000 platform (Illumina, USA) to generate paired-end reads. The initial quality filtering of paired-end Fastq files was performed using Fastp (v0.20.1) under stringent criteria: low-quality bases (Q<20) were trimmed from read termini, low-complexity sequences were eliminated, and reads shorter than 35 bp were excluded. The filtered reads were aligned against the *Homo sapiens* reference genome (GRCh38) using Bowtie2 (v2.4.2) to remove host-derived sequences (Langmead and Salzberg, 2012). Subsequently, species annotation was performed by comparing the sequencing data to the NCBI nr database, and functional annotation was performed by comparing the sequencing data to the KEGG database.

### Metabolomics analysis

Metabolomics analysis was performed on an UHPLC-Q Exactive HF-X (Thermo Fisher Scientific, Germany). Variable importance in projection (VIP) scores were used to identify candidate metabolites. MS/MS fragments were structurally characterized using MassFragment™ software (Waters Corporation). Putative biomarkers were annotated by interrogating the Metlin, HMDB, and ChemSpider databases. For the metabolomic analysis, participants in the disease group were randomly selected from the original cohort, and healthy controls (HC) were chosen to match potential confounding factors such as sex, age, and body mass index (BMI). The raw data of these participants were converted into MzXML files using ProteoWizard MSConvert and subsequently imported into XCMS software for further analysis.

### Network analysis

Microbial co-occurrence networks were constructed using 16S rRNA gene sequencing data according to a method previously described (Hong et al., 2024). The network topology was characterized by quantified parameters (nodes, links, average clustering coefficient, connectedness) and interaction properties (positive and negative link ratios), and by modularity and keystone taxa. The microbiota-metabolite association network was constructed using the R package MetaNet (v0.13) based on Spearman correlation coefficients. Only interactions between differentially abundant bacteria and differential metabolites were retained. Parameter settings for the c_net_build functions were as follows: r_thres = 0, p_thres = 0.05, and delete_single = TRUE. Network modules were subsequently identified using the module_detect function, using a minimum module size threshold of 10 nodes (n_node_in_module = 10) and removing unassigned nodes (delete = TRUE) as described in Mu et al (Wenjie et al., 2024).

### Statistical analysis and visualization

Results are presented as mean ± SEM. Statistical comparisons were performed with one-way ANOVA (Tukey’s *post hoc*) or two-tailed Student’s *t*-test using GraphPad Prism 6.01 (GraphPad Software). Significance thresholds were as follows:* *P* < 0.05, **P < 0.01, and ***P < 0.001. Alpha diversities (ACE, observed species, Shannon, and Chao1 indices) were computed, and principal coordinates analysis (PCoA) based on Bray–Curtis distances was performed using the Tutools platform and visualized with ImageGP. Differentially abundant microbial features were identified through linear discriminant analysis effect size (LEfSe) with a linear discriminant analysis (LDA) score > 2 and *P* < 0.05. To assess associations between parasite infection abundance, microbial features, and KEGG ortholog groups, pairwise Spearman’s rank correlations were calculated. Correlations with |ρ| < 0.7 and *P* > 0.05 were excluded, and *P*-values were adjusted for false discovery using the Benjamini–Hochberg method (Hmisc package, R v4.2.2). Network analysis and visualization were conducted in Cytoscape (v3.9.1).

## Results

### Composition of gut microbiota in patients with whipworm infection

A total of 1,012 fecal samples were collected in this study, with over 160 samples re-examined. Ultimately, 75 whipworm-positive samples were identified, resulting in a whipworm infection rate of 7.4% (75/1012) among the rural residents of Lanshan District. Fifteen whipworm-positive cases with a mean eggs per gram of stool (EPG) of 552.0 (range, 24.0–5856) and fifteen healthy controls were included in the final study (**Table 1**).

**Table 1:** The metadata of the cohort in this study.

Variable	Healthy controls	Infected patients	P value
Sex			
Female	7 (46.7%)	6 (40.0%)	0.136
Male	8 (53.3%)	9 (60.0%)	
EPG			
Mean SD	0	552.0 (1475.5)	
Median [Min,Max]	0	144 [24.0, 5856]	<0.0001
BMI			
Mean (SD)	23.3(3.9)	22.6 (2.7)	0.5
Median [Min,Max]	22.92 [15.9, 26.0]	22.2 [18.0,26.9]	

**Figure 1:**
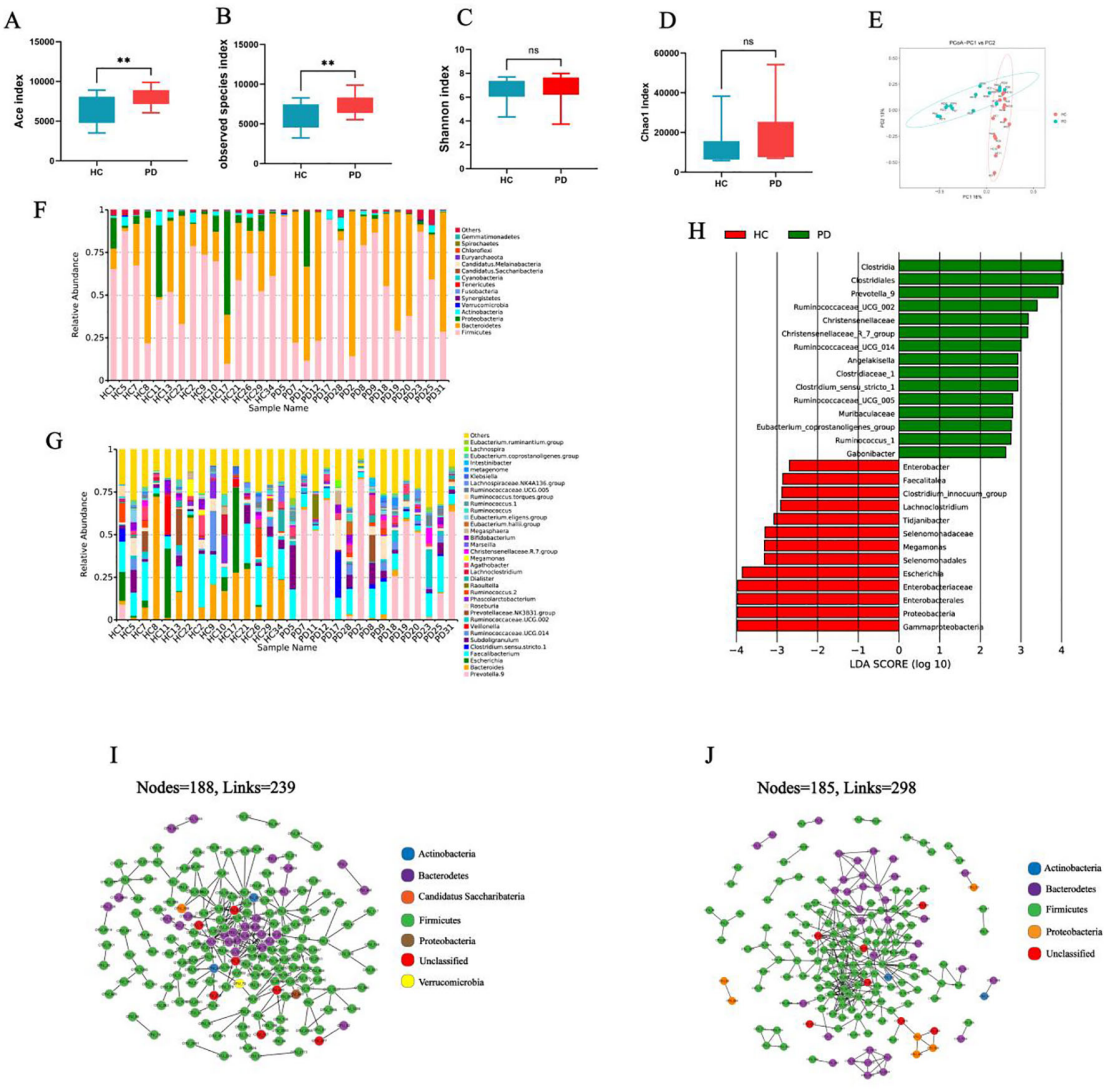
Differences in the gut microbes of the whipworm-infected group (parasitic disease, PD) and healthy controls (HC). Comparative analysis of ACE **(A)**, observed species **(B)**, Shannon **(C)**, and Chao1 **(D)** indices among different groups. **(E)** PCoA analysis based on weighted UniFrac distances to identify microbial composition changes. Top 15 species at the phylum level **(F)** and top 35 at the genus level **(G)** among the different groups. **(H)** LEfSe species counts between PD and HC groups. Co‐occurrence network analysis illustrating potential interactions between bacteria of the PD **(I)** and HC groups **(J)**. **P< 0.01.

The complexity of the microbial communities was quantified using α-diversity indices (observed species, ACE, and Shannon) based on 16S rRNA gene sequencing data. A significant increase was found in both the observed species index (t=2.95, P=0.006) (**Figure 1A**) and ACE index (t=2.93, P=0.007) (**Figure 1B**), with statistically significant upregulation in the whipworm-infected group (parasitic disease, PD) relative to healthy controls (HC). However, no significant change was found in the Shannon index (t=1.291, P=0.21) (**Figure 1C**) or Chao1 index (t=0.03, P=0.97) (**Figure 1D**), suggesting that higher species richness and species evenness and overall diversity remained unaffected. A beta diversity assessment using PCoA based on weighted UniFrac distances revealed significant separation between the microbial communities of the PD group and HC group (**Figure 1E**).

To investigate the changes in the gut microbiota of patients with whipworm infection, we examined the community structure of the microbiota in patients with whipworm infection and in healthy controls. Firmicutes, Bacteroidetes and Proteobacteria were the most abundant phyla. Compared to uninfected individuals, patients with whipworm infection showed decreased Proteobacteria and increased Firmicutes (**Figure 1F**). At the genus level, the abundance of *Prevotella 9* increased in patients infected with whipworm, while the levels of *Bacteroides*, *Escherichia*, and *Klebsiella* decreased (**Figure 1G**). Our results indicated that whipworm infection altered the gut microbiota of the patients. We used LEfSe to analyze the intestinal microbial species in each group. *Clostridia, Prevotella* 9, *Ruminococcaceae* UCG-002, and the *Christensenellaceae* R-7 group were enriched in the PD group, while *Escherichia, Megamonas, Tidjanibacter*, and the *Clostridium innocuum* group were enriched in the HC group (**Figure 1H**).

Co-occurrence networks were used to delineate whipworm infection-induced changes in microbial interaction dynamics within the gut ecosystem. As shown in **Figures 1I, J**, the co-occurrence networks of the HC group were clearly more complex than those of the PD group, indicating that whipworm infection decreased the network complexity of gut microbiota.

### Metagenomic sequencing analysis

**Figure 2:**
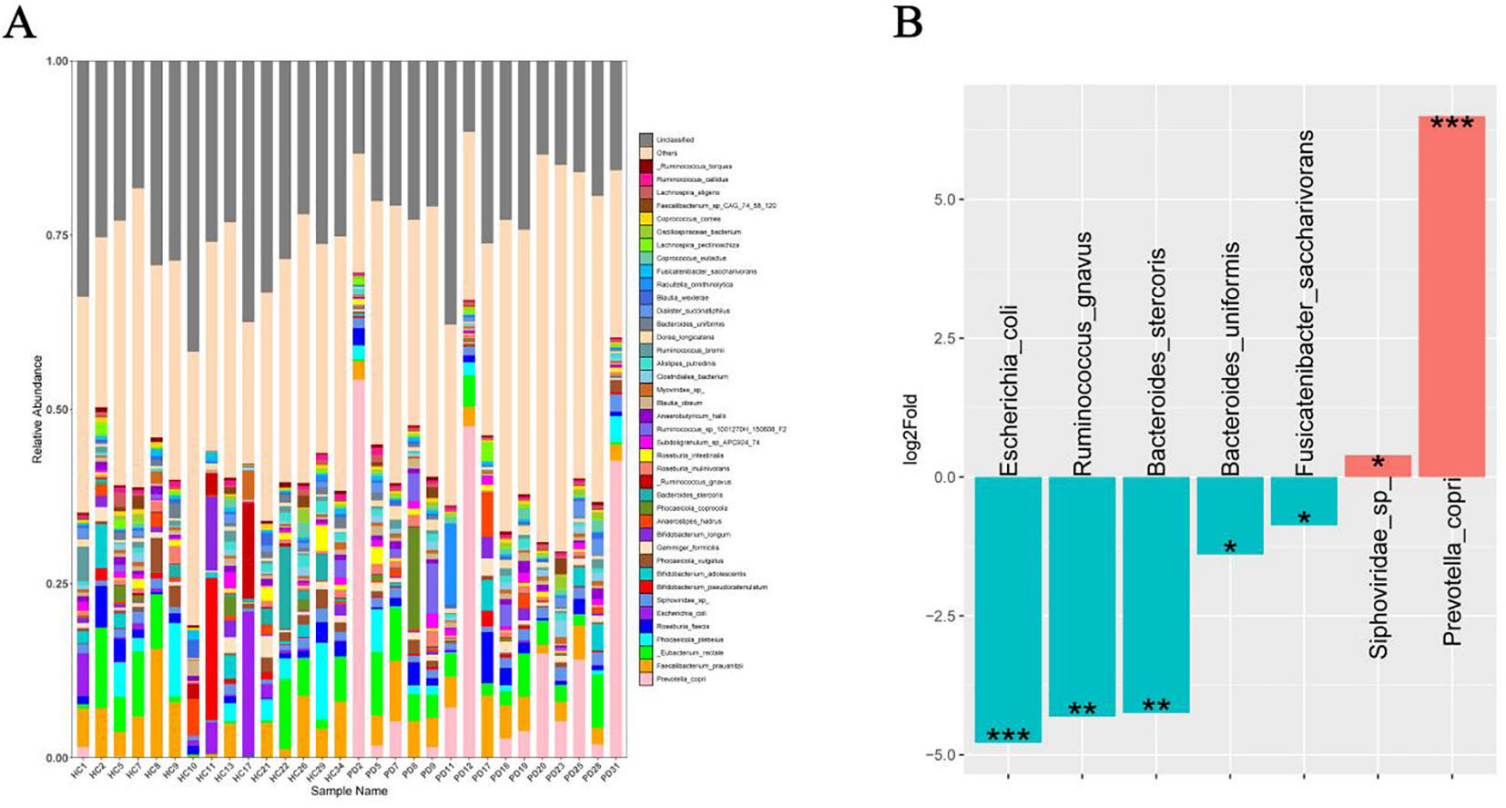
Metagenomic analysis of gut microbiota of the PD and HC groups. **(A)** Species comparison analysis of the top 40 species among the different groups. **(B)** Fold changes of PD-enriched and PD-depleted species. *P < 0.05, **P < 0.01, and ***P < 0.001.

Metagenomic sequencing analysis revealed distinct microbial enrichment patterns between the PD and HC groups (**Figure 2A**). The abundance of *Prevotella copri* (*P*=0.001) and *Siphoviridae* sp. (*P*=0.014) were significantly increased in the PD group (**Figure 2B**). In the HC group, there was marked upregulation of *Bacteroides stercoris* (*P*=0.001), *B. uniformis* (*P*=0.002), *Escherichia coli* (*P*=0.001), *Fusicatenibacter saccharivorans* (*P*=0.001), and *Ruminococcus gnavus* (*P*=0.001) (**Figure 2B**).

**Figure 3:**
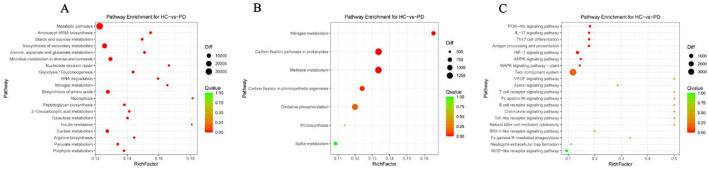
Predicted functional potential of the gut microbiota in the fecal microbiota. **(A)** Functional comparative analysis of the top 20 metabolites of the PD group and HC group identified by the KEGG database. **(B)** Metabolic capacity (KEGG level 2, energy metabolism). **(C)** Immune-related pathways (KEGG level, immune signaling).

The functional annotation using the KEGG database revealed 367 enriched pathways between the PD and HC groups in the metagenome with metabolism emerging as the most abundant functional category in level 1. This suggested a crucial and far-reaching role of metabolism in the overall biological functions represented in the metagenome. The level 2 categories within metabolism predominantly included global and overview maps, carbohydrate metabolism, amino acid metabolism, energy metabolism, metabolism of cofactors and vitamins, glycan biosynthesis and metabolism, nucleotide metabolism, and lipid metabolism (**Supplementary Table 1**). A deeper analysis of the specific metabolic processes showed enrichment in pathways critical to core biochemistry, such as metabolic pathways, biosynthesis of secondary metabolites, biosynthesis of amino acids, and central carbon processing pathways, such as carbon metabolism, pyruvate metabolism, carbon fixation pathways in prokaryotes, and carbon fixation in photosynthetic organisms (**Figure 3A**). Additionally, pathways related to energy generation (microbial metabolism in diverse environments, energy metabolism), nitrogen utilization (nitrogen metabolism), methane cycling (methane metabolism), and biosynthesis (porphyrin metabolism) were notably enriched (**Figure 3B**). These results highlighted that the microbial community was highly active and complex, and involved in diverse and interconnected metabolic functions. In addition to the active metabolic pathways, immune-related pathways at level 1 included infectious disease, viral; infectious disease, parasitic; infectious disease, bacterial; and immune disease (**Supplementary Table 1**). Similarly, the top enriched immune pathways comprised PI3K-Akt signaling, IL-17 signaling, HIF-1 signaling, Th17 cell differentiation, antigen processing and presentation, AMPK signaling, and the two-component system (**Figure 3C**).

### Metabolome analysis

**Figure 4:**
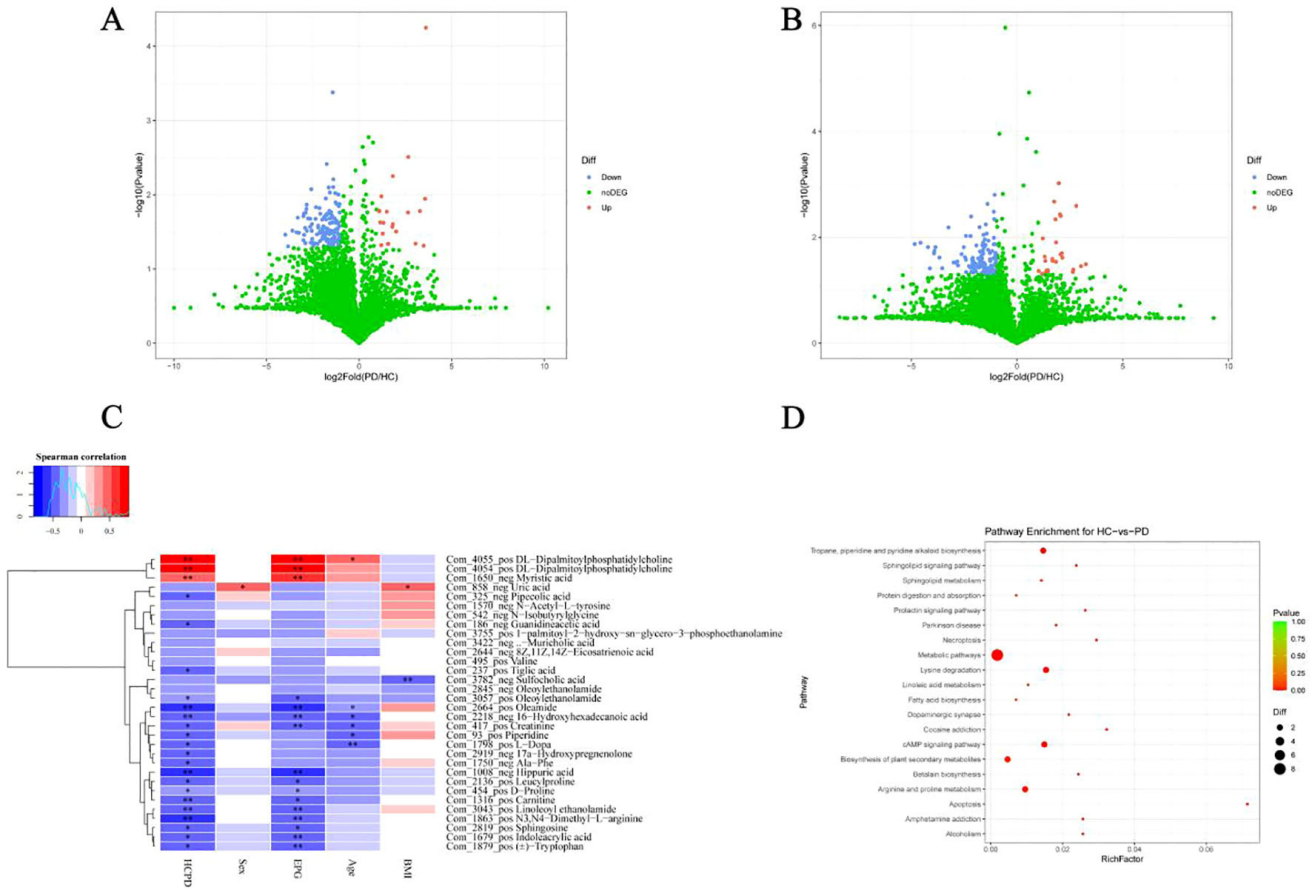
Differentially abundant metabolites in the feces between the PD and HC groups. Volcano maps of differential metabolites under positive ion mode **(A)** and negative ion mode **(B, C)** Heatmap of the Spearman correlation analysis between differential metabolites and variable factors (infection status, sex, EPG, age and BMI). **(D)** Functional comparative analysis of metabolic components in the KEGG database (top 20). *P < 0.05 and **P < 0.01.

The fecal samples of the PD and HC groups were used for metabolomic analysis by LC/MS. Metabolomic profiling identified significant differences between the PD and HC groups. In positive ion mode, 158 differentially abundant metabolites were detected, of which 21 metabolites had increased concentrations and 137 had decreased concentrations in the PD group (**Figure 4A**; **Supplementary Table 2**). Similarly, negative ion mode analysis revealed 154 dysregulated metabolites in the PD group, of which 27 were upregulated and 127 were downregulated (**Figure 4B**; **Supplementary Table 3**). Correlation heatmaps revealed potential relationships between whipworm infection and the metabolites. Myristic acid and DL-dipalmitoylphosphatidylcholine had strong positive correlations with whipworm infection (HCPD) and EPG. Oleoylethanolamide, 16-hydroxyhexadecanoic acid, creatinine, hippuric acid, leucylproline, D-proline, carnitine, linoleoyl ethanolamide, N3,N4-dimethyl-L-arginine, sphingosine, indoleacrylic acid, and tryptophan had strong negative correlations with whipworm infection (HCPD) and EPG (**Figure 4C**). A KEGG enrichment analysis of the differential metabolites revealed the metabolic pathway changes associated with whipworm infection. The dysregulated metabolites were predominantly enriched in five key pathways: metabolic pathways; tropane, piperidine, and pyridine alkaloid biosynthesis; lysine degradation; cAMP signaling pathway; and arginine and proline metabolism. These metabolic perturbations collectively indicated physiological response mechanisms to whipworm infection in the elderly (**Figure 4D**).

### Microbiota-metabolite correlation analysis

**Figure 5:**
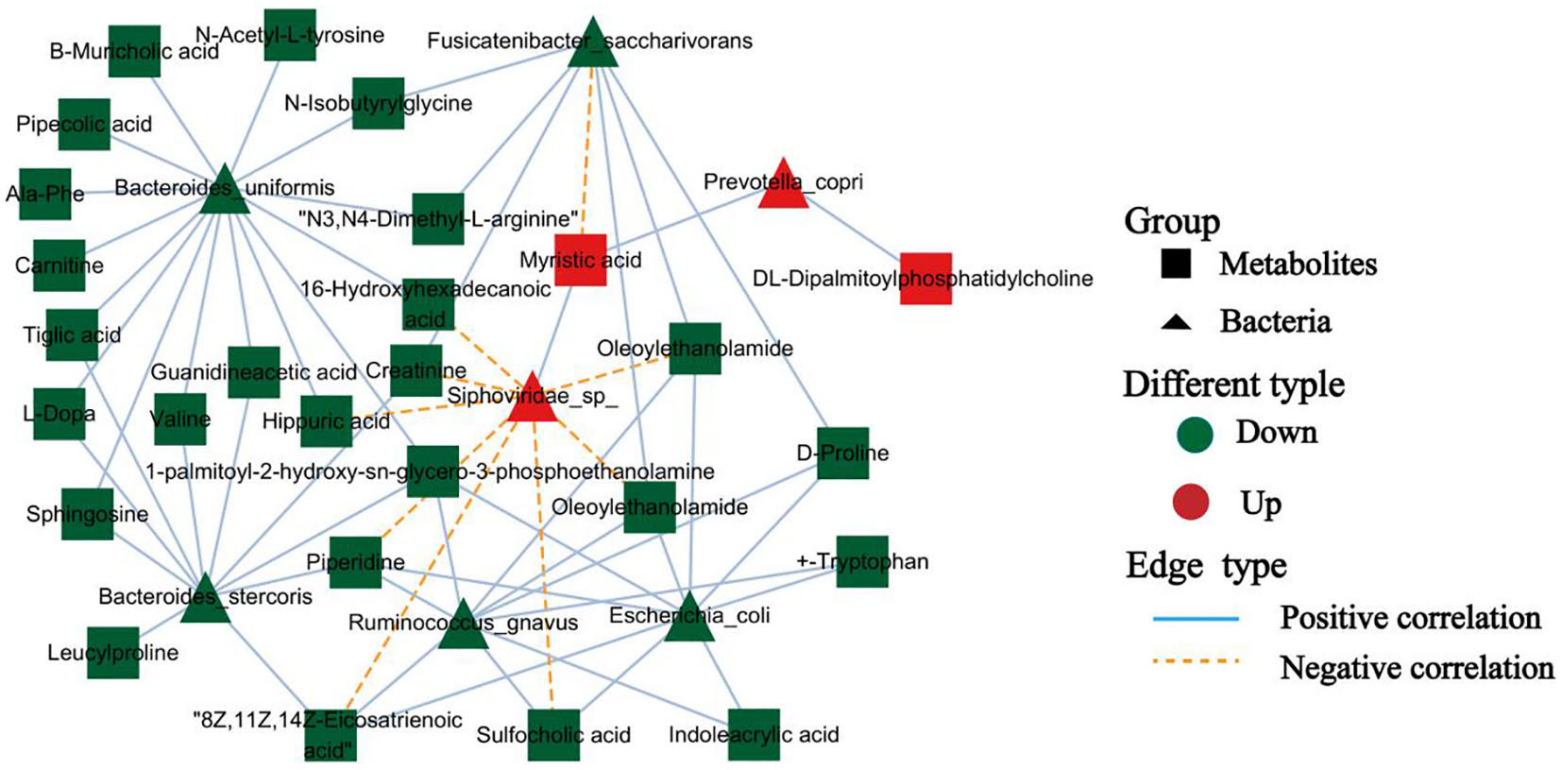
Microbiota-metabolite interaction network. Nodes represent bacteria (triangle) and metabolites (square), and edges indicate positive (blue) or negative (orange) correlations.

Because the physiological effects of the gut microbiota on the host are often mediated by a complex host–microbe metabolic axis involving numerous biochemical reactions, we next analyzed the associations between the abundance of specific bacterial species and metabolites related to whipworm infection. We found that *P. copri* and *Siphoviridae* sp. showed positive correlations with the upregulated levels of the fecal metabolite myristic acid. Interestingly, the downregulated metabolites, such as 16-hydroxyhexadecanoic acid, creatinine, hippuric acid, and oleoylethanolamide, correlated with a reduced abundance of *B. stercoris*, *B. uniformis*, *E. coli*, *F. saccharivorans*, and *R. gnavus* (**Figure 5**). These findings highlighted a clear connection between changes in gut metabolites and shifts in the gut microbiota, particularly the increase in *P. copri* and *Siphoviridae* sp. during infection.

## Discussion

The gut microbiota is crucial in host physiology and health, and its study has garnered significant attention. In this study, we provided evidence of changes in both the gut microbiota composition and metabolic profiles associated with whipworm infection in the elderly population of Lanshan District, Shandong Province, China. Our findings elucidated the effects of whipworm infection on the gut microbial composition, functional potential, and metabolic profiles, contributing to a better understanding of the complex interplay between helminth infection and host health, particularly in elderly individuals. Notably, this research highlights the effects of these infections in elderly individuals, a group that is underexplored in studies of soil-transmitted helminths.

We found a significant increase in the alpha diversity (ACE index and observed species index) among whipworm-infected individuals compared to healthy controls (**Figure 1**). Numerous studies investigating the relationship between helminth infections and microbial diversity have yielded inconsistent findings, with several investigations reporting divergent results. For example, a systematic review and meta-analysis by Kupritz et al. found both an increased and unchanged microbial diversity in helminth-infected individuals. Conversely, a study on Ethiopian children infected with whipworm and other soil-transmitted helminths reported a significantly reduced microbial diversity compared to uninfected children (Taye et al., 2024). Notably, Cupper et al. reported that patent infections of *T. trichiura* and *Ascaris lumbricoides* were not significantly associated with changes in the gut microbiota alpha diversity of Ecuadorian school children (Cooper et al., 2013). These discrepancies across studies may be attributed to heterogeneity in helminth species, infection burden, host age, nutritional status, geographic location, and methodological differences in microbiome profiling. Our findings, which aligned with the increased microbial richness reported in some studies, suggest a potentially species-specific or context-dependent effect of *T. trichiura* on gut microbial diversity. Further studies using longitudinal designs and standardized methodologies are warranted to elucidate the mechanisms underlying these associations.

Chronic whipworm infection has emerged as an important pathogenic factor that disrupts intestinal immune homeostasis. The evidence suggests that persistent colonization by *Trichuris* spp. compromises intestinal mucosal integrity, thereby facilitating the translocation of proinflammatory pathobionts across the epithelial barrier (Wammes et al., 2014). This breach in barrier function initiates and sustains aberrant immune activation; chronic *Trichuris* infection is therefore a modifiable risk factor that can lead to the onset and progression of enteritis and inflammatory bowel disease (IBD) (Schlmerich, 2013). Mechanistically, macrophages isolated from *Trichuris*-infected hosts exhibited heightened sensitivity to lipopolysaccharide stimulation, adopting a hyperresponsive phenotype indicating immune priming. These immunomodulatory events culminated in a chronic inflammatory phase characterized by a dysregulated T-cell landscape with concurrent activation of Th1, Th2, and Th17 cytokine pathways (Li et al., 2011). Notably, the abundance of Firmicutes increased, while the abundance of Proteobacteria decreased in the whipworm-infected group, consistent with previous reports indicating that helminth infection shifted the microbiota composition (Kupritz et al., 2021). Specifically, the genus *Prevotella 9* was significantly associated with whipworm infection, while *Bacteroides*, *Escherichia*, and *Klebsiella* were found to be depleted (**Figures 1G, H**). Further metagenomic sequencing analysis at the species level revealed distinct microbial signatures, with *P. copri* and *Siphoviridae* sp. significantly enriched, whereas species associated with gut health, including *B. stercoris*, *B. uniformis*, *F. saccharivorans*, and *R. gnavus*, were significantly depleted in the PD group (**Figure 1A**). These observations were consistent with previous reports linking helminth infections to a transition toward a *Prevotella* enterotype (Rosa et al., 2021; Taye et al., 2024; Wenjie et al., 2024; Zhou et al., 2023). *P. copri*, the most prominent and widespread member of the *Prevotella* genus, is frequently found in the gut microbiota of non-Western populations and has been associated with dietary fiber intake (Ahmed, 2024). Certain *P. copri* strains were found to promote intestinal inflammation by disrupting bile acid metabolism and overproducing proinflammatory metabolites. The complexity of Prevotella is particularly evident in the context of autoimmune diseases, such as rheumatoid arthritis (RA), in which the *P. copri* abundance correlated with disease susceptibility and severity (Pianta et al., 2017; Scher et al., 2013; Wang et al., 2025). Notably, a *P. copri* strain isolated from RA patients (RA-type *P. copri*) was found to exacerbate collagen-induced arthritis in mice through a diet-microbe-metabolite axis (Jiang et al., 2022). *Prevotella* species have also been implicated in dendritic cell activation and Th17 polarization through lipopolysaccharide-like signaling, which elicited chronic inflammatory responses in both gut and extraintestinal tissue. For example, mucosal antigen-presenting cells exposed to *Prevotella*-enriched environments upregulated IL-23A, IL-6, IL-1α, and IL-1β expression, key mediators of Th17 differentiation (Larsen, 2017). This was corroborated in models of periodontal disease and vaginal inflammation, in which *Prevotella* colonization recruited neutrophils via Th17 activation, contributing to epithelial damage and barrier dysfunction (Yang et al., 2019). These observations are consistent with our metagenomic functional profiling results (**Figure 3C**), which indicated significant enrichment of proinflammatory signaling pathways in the infected group, including Th17 cell differentiation and IL-17 (Jiang et al., 2023), PI3K-Akt, and AMPK signaling pathways (Wang et al., 2022). These pathways are known to drive chronic inflammation and promote proinflammatory cytokine production. The marked depletion of *B. stercoris*, *B. uniformis*, *R. gnavus*, and *F. saccharivorans* observed in chronic *Trichuris* infection suggests a broad suppression of key microbial taxa that maintain intestinal immune and metabolic homeostasis (**Figure 2A**). Notably, *B. stercoris* and *F. saccharivorans* were implicated in the production of short-chain fatty acids, which are known to promote regulatory T-cell responses and suppress pro-inflammatory pathways, including NF-κB and MAPK signaling (Takada et al., 2013; Woo et al., 2024; Yang et al., 2021, Yang et al., 2022). Supplementation with *B. uniformis* ameliorated dextran sulfate sodium-induced colitis in mice, in part by modulating bile acid metabolism and inhibiting Th17 differentiation and ferroptosis (Yan et al., 2023). The reduced abundance of these strains in the *Trichuris*-infected gut may tip the immune balance toward chronic inflammation. Collectively, these findings highlighted that chronic *Trichuris* infection perturbed gut homeostasis and actively contributed to the initiation and perpetuation of IBD. Importantly, these insights raise the possibility of targeting specific microbiota components or metabolic pathways as therapeutic interventions. Microbiota-based therapies that modulate *Prevotella*-dominated enterotypes, restore commensal taxa, or dampen Th17-polarizing microbial signals may provide promising treatments against *Trichuris* infection. Future studies incorporating fecal microbiota transplantation, probiotics, or bile acid-modulating agents should explore the therapeutic potential of correcting helminth-associated dysbiosis in chronic inflammation.

The relationship between gut microbiota and metabolite profile changes is particularly noteworthy. We identified a strong association between *P. copri* and increased levels of myristic acid (MA), suggesting that specific microbial taxa may contribute to the production of metabolites involved in host metabolism (**Figure 5**). In contrast, the upregulated species in the healthy controls (*B. stercoris*, *B. uniformis*, *R. gnavus*, and *F. saccharivorans*) were associated with a decrease in metabolites linked to anti-inflammatory effects, such as oleoylethanolamide (OEA), linoleoyl ethanolamide (LEA), tryptophan, creatinine, hippuric acid, D-proline, and carnitine (**Figure 5**). Emerging evidence suggests that elevated MA levels may actively influence intestinal and systemic inflammatory processes, particularly in certain diseases or infections. Notably, high MA intake was identified as an independent risk factor for clinical relapse in ulcerative colitis patients undergoing aminosalicylate therapy (Barnes et al., 2017). The pro-inflammatory effect of MA appears to extend beyond the gut, as dietary MA supplementation in obese models exacerbated adipose tissue inflammation and systemic insulin resistance at the cellular level. MA is a key structural component in the lipidation (N-myristoylation) of signaling proteins, a modification that modulates immune cell activation thresholds. For example, MA incorporation promoted STING-dependent interferon responses and autophagy pathways in macrophages (Jia et al., 2023). Given these multifaceted pro-inflammatory properties, MA upregulation during *Trichuris* infection may have deleterious consequences for gut barrier function, immune tolerance, and disease progression in chronic inflammatory conditions. Future work should explore whether targeting MA biosynthesis or signaling pathways can mitigate post-infectious inflammatory sequelae, particularly in patients with preexisting intestinal disorders. OEA, a lipid mediator synthesized in enterocytes, is vital in nutrient sensing, satiety, and energy balance (De Filippo et al., 2023). Recent studies showed that OEA exerted potent anti-inflammatory effects by suppressing NF-κB activation through peroxisome proliferator-activated receptor alpha (PPAR-α) signaling (Otagiri et al., 2020). Furthermore, OEA administration was shown to reshape the gut microbial community toward a less inflammatory profile (De Filippo et al., 2023). Similarly, levels of LEA, another fatty acid ethanolamide reported to have anti-inflammatory properties, were decreased. In animal models, LEA deficiency has been associated with microbiota disturbances and inflammation-driven lipid dysregulation (Song et al., 2024; Zhou et al., 2024). Tryptophan and its microbial catabolites, such as indoleacrylic acid, are integral to the gut–immune axis, enhancing barrier function and suppressing proinflammatory macrophage polarization through aryl hydrocarbon receptor (AhR) signaling (Jiang et al., 2024; Song et al., 2024; Zhou et al., 2024). Microbiota-driven metabolism of tryptophan also affects serotonin biosynthesis and mucosal immunity (Agus et al., 2018). These findings suggest that whipworm infection induces a dysbiotic microbial state that alters both the composition of gut microbiota and the metabolites they produce, potentially exacerbating inflammation and impairing the host’s metabolic processes. Future studies should investigate whether restoring or supplementing these metabolites can counteract post-infection sequelae and support gut recovery in helminth-endemic populations.

## Conclusion

This study provides novel insights into the effects of whipworm infection on the gut microbiota and metabolic profile in an elderly population. Our findings revealed that whipworm infection induced significant changes in both the composition and functional potential of the gut microbiota and produced metabolic perturbations that may contribute to dysregulated host immune and metabolic pathways. Metagenomic and metabolomic analyses highlighted distinct microbial signatures and metabolic pathways associated with whipworm infection. Notably, increased levels of pro-inflammatory metabolites, including myristic acid, were observed in the PD group, which may have exacerbated intestinal inflammation and disrupted gut barrier integrity. Moreover, anti-inflammatory metabolites such as oleoylethanolamide and tryptophan-related compounds were depleted, further underscoring the potential of *T. trichiura* infection to impair host immune tolerance and metabolic homeostasis. These findings emphasize the importance of considering the complex interactions between helminth infection, gut microbiota, and host immunity in older adults. These interactions appear to crucially modulate both local and systemic inflammatory responses, which may have long-term health implications, particularly in populations at risk for chronic inflammatory diseases. Future studies should further explore the potential of microbiota-based interventions, such as fecal microbiota transplantation or targeted probiotics, to mitigate the inflammatory sequelae of chronic whipworm infections. Additionally, contextual factors such as environmental exposure, socioeconomic status, and healthcare access must be addressed to develop effective strategies to combat whipworm infections and their associated health consequences in aging populations. This work lays the foundation for future investigations into the role of gut microbiota in modulating host responses to parasitic infections, with the aim of identifying novel therapeutics to improve health outcomes for vulnerable populations.

## Data Availability

The datasets presented in this study can be found in online repositories. The names of the repository/repositories and accession number(s) can be found in the article/**Supplementary Material**.
